# Exploring citizens’ preferences for the temporal effectiveness of urban nature-based solutions through participatory GIS

**DOI:** 10.1038/s42949-025-00229-5

**Published:** 2025-07-01

**Authors:** Alessia Chelli, Christopher M. Raymond, Silviya Korpilo, Davide Geneletti

**Affiliations:** 1 Department of Economics and Management, University of Trento, Trento, Italy; 2 Department Civil, Environmental and Mechanical Engineering, University of Trento, Trento, Italy; 3 Helsinki Institute of Sustainability Science, University of Helsinki, Helsinki, Finland; 4 Ecosystems and Environment Research Program, Faculty of Biological and Environmental Sciences, University of Helsinki, Helsinki, Finland; 5 Department of Economics and Management, Faculty of Agriculture and Forestry, University of Helsinki, Helsinki, Finland; 6 Digital Geography Lab, Department of Geosciences and Geography, University of Helsinki, Helsinki, Finland; 7 Helsinki Institute of Urban and Regional Studies, University of Helsinki, Helsinki, Finland

**Keywords:** Environmental social sciences, Sustainability

## Abstract

Temporal dynamics remain an understudied aspect of nature-based solutions (NBS) literature, particularly in relation to public preferences. This study introduces the concept of NBS temporal effectiveness, defined as the ability of NBS to provide co-benefits over short, medium, and long terms with varying intensities, as perceived by urban residents. Specifically, we investigated the potential conflicts in the temporal effectiveness in Trento, Italy, through a public participation GIS (PPGIS) approach (*n* = 286). Our findings reveal that the intensity and directionality of conflict change across short, medium and long-term NBS, respondent groups, and the spatial context of implementation. Contrary to the common perception that immediate solutions are always preferred, NBS with long-term effectiveness can gain significant public support, particularly when they demonstrate greater benefits than short-term alternatives. This study contributes to the NBS literature by being the first to explore the concept of temporal effectiveness and its implications for public preferences, demonstrating that considerations regarding temporalities must be made when planning NBS interventions, as conflicts may arise in relation to interventions with different temporal effectiveness.

## Introduction

In the urban context, nature-based solutions (NBS) have gained increasing attention due to their potential to address societal challenges and enhance resilience against climate change^1–3^. NBS can provide a range of different benefits, depending on the type and scale of the intervention, and the characteristics of the local context^4,5^.

NBS effectiveness is assessed through qualitative and quantitative indicators, which can be categorized as follows: environmental indicators, such as the tones of carbon stored or the percentage increase in species diversity; human health and well-being indicators, such as the reduction in mortality rates due to air pollution; citizen participation indicators, such as the proportion of citizens actively involved in implementing green projects; and transferability indicators, which measure aspects related to the transfer and monitoring of projects^6^. The selection of indicators to evaluate NBS effectiveness depends on the goal and type of intervention, as well as the geographical and temporal scale^7^. Indeed, NBS are living and dynamic systems, and the ecosystem services (ES) they provide —i.e. the direct and indirect benefits that ecosystems provide to human well-being— can change over time^8,9^. Factors such as the type of ecosystem used, the vegetation composition and growth time^10^, and the location influence NBS implementation and the socio-ecological processes they support^11,12^. The interaction between these factors and external attributes—including biodiversity, local climate, topography, land cover, human activities, and management practices— may further affect the temporal dynamics of ES flows from NBS^13,14^. This means that certain types of NBS may take many years to achieve their full range of benefits, and different ES can emerge at various stages^4,15^. Some ES may manifest immediately following NBS implementation, while others may only become apparent in the long run^16^. This highlights the significant role that time plays in the success of NBS, as they may not always provide an instant solution to environmental or social challenges^17^.

In general, three main patterns can be identified in ES temporal dynamics: linear dynamics – continuous increase or decrease in ES supply and demand; periodic dynamics – linear trends with oscillations around a mean value, and irregular events – occasional disturbances in ES supply and demand^18^. Despite the recognition of the temporal dynamics of NBS and their co-benefits^7,18,19^, few studies have analyzed how the co-benefits of NBS evolve over time. Most existing NBS frameworks do not incorporate dynamic simulations^20^, and NBS effectiveness is often assessed at a single point in time, without accounting for growth and development^21,22^. Moreover, analyses of NBS typically assume that both the solutions themselves and the conditions under which they operate (e.g., climate, urbanization) remain unchanged^23^. This assumption represents a significant gap in the literature^6^.

Similarly, the ES literature has not fully addressed temporal dynamics. A review by Rau et al.^24^ reveals that only 2% of the ES literature considers temporal dynamics. Likewise, a review by Obiang Ndong et al.^22^, which examined 103 studies, found that only 6% investigated the spatio-temporal relationships of ES. In this context, short-term ES dynamics are typically analyzed using experimental data and field samples/observations, while long-term studies often rely on remotely sensed data, secondary data, or simulations^18,25,26^. Overlooking which NBS are more effective in the short or long term, as perceived by residents, constitutes a barrier to their implementation^6,20^. This issue becomes particularly evident when comparing NBS with grey infrastructure, which often appears to deliver quicker benefits, even though this is not always the case^9^. This limitation is also reflected in the economic evaluations of NBS. A recent review of cost-benefit analyses on urban NBS showed that most studies assume the full delivery of co-benefits within the first year of implementation, and maintain this assumption throughout the entire lifecycle of the infrastructure, affecting the accuracy of the economic evaluations^27^.

Previous studies have suggested to enhance NBS impact assessment and monitoring methodologies to reflect changes in the effectiveness of co-benefits over time, emphasizing the importance of considering the time required for these benefits to materialize^7^. A report from the European Union-funded Nature4Cities project^28^ provides a factsheet for each type of NBS, detailing both the benefits and co-benefits they offer and their respective maturation periods. For example, interventions like urban farms, rain gardens, or swales can become fully operational within relatively short timeframes (usually one to five years), thus delivering immediate benefits. Conversely, tree-based solutions—such as urban forests or street trees—may take up to a decade to mature, but in this context, that longer timescale yields more substantial advantages than short-term interventions, including enhanced carbon sequestration, biodiversity gains, and improved flood mitigation. It is important to note, however, that these temporal dynamics do not apply uniformly to every NBS, and a longer path to full effectiveness does not necessarily translate into greater benefits in every case.

Building from the two fundamental concepts of NBS effectiveness and temporality, we introduce the concept of “NBS temporal effectiveness”. This term refers to the time required for an NBS to deliver its full range of benefits at maximum capacity and is founded on a trade-off between the timing of benefits and their magnitude: the more immediate the benefits, the lower their potential magnitude. The classification is structured as follows:

- NBS with short-term effectiveness: this category includes NBS that delivers limited co-benefits that reach their maximum effectiveness within one year from the implementation.
- NBS with medium-term effectiveness: this category includes NBS that deliver enhanced co-benefits compared to short-term NBS, achieving their full effectiveness within 3–5 years of implementation.
- NBS with long-term effectiveness: this category includes NBS that deliver enhanced co-benefits compared to medium-term NBS, which can take up to 10 years to achieve their full effectiveness.

We hypothesize that short-term NBS interventions primarily involve vegetated components such as grassy meadows and annual plants, medium-term interventions include a combination of annual plants and fast-growing trees, and long-term NBS involve trees and perennial plants that take more time to mature but ultimately yield greater benefits. Finally, it is important to recognize that the same NBS can generate different benefits at varying times and magnitudes, depending on factors such as the species involved, the vegetation’s maturity, local context, and prevailing environmental conditions^29,30^. This classification is not intended as a framework for categorizing NBS types according to benefit timing and effectiveness, as such a framework would require dedicated monitoring and simulation studies^31,32^, which are scarce in the literature. The proposed classification should be viewed primarily as a guide to investigate how the time lag in generating co-benefits affects support for NBS as well as the extent to which citizens are willing to trade immediate but lower benefits for delayed but higher benefits and vice versa, and must be adapted to each specific context, since local settings and environmental conditions can influence the temporal effectiveness of the chosen NBS.

Despite the recognition of these dynamics within the NBS literature, little research has conceptualized and assessed the temporality of NBS co-benefits as perceived by residents. Previous studies have identified both the delayed realization of benefits^33,34^ and the associated knowledge gap^35^ as barriers to NBS implementation. They have also stressed the need to explore public preferences regarding the temporality of NBS to enhance their social acceptance^36^, and to include temporal variability of co-benefit in the economic assessment of NBS^27^. However, temporal dynamics of NBS remain understudied. This represents a critical knowledge gap, as understanding these dynamics and learning how to leverage them to address citizens’ needs can improve the effectiveness of NBS planning. For instance, it can help prevent mismatches between expected and actual benefits, which might otherwise undermine public support for NBS.

While the temporal effectiveness of NBS remains generally understudied, there is an even greater lack of research examining public preferences and support for NBS in relation to their temporal effectiveness using Public Participation GIS (PPGIS). PPGIS is a participatory mapping approach that enables participants to locate and describe features on a map, integrating their knowledge, values, and preferences into collaborative spatial decision-making, and can thus be regarded as a type of planning support system^37–39^. Numerous studies demonstrate the versatility of PPGIS in the context of NBS and land-use planning by capturing social values and cultural ES^40–43^, identifying meaningful places for NBS planning^44^, addressing environmental justice and social integration^45–47^, co-designing interventions^48^, and evaluating support for environmental policies and management^49,50^. PPGIS has also been used to identify potential synergies and conflicts in land use^51,52^, further underscoring its relevance as a decision-support tool in sustainable urban and regional planning. Although the use of PPGIS in scientific research has grown over time, planning professionals still exhibit some reluctance toward participatory approaches, often perceiving expert opinions as more reliable^53^. As emphasized by Ives et al.^42^, future empirical research employing PPGIS should not only address scientific and theoretical considerations but also explore its practical application in landscape planning.

This study is positioned within this context and presents a PPGIS study in the city of Trento, located in northeastern Italy, with the following objectives:

- To identify clusters of respondents based on their preferences for the temporal effectiveness of NBS.
- To spatially identify the potential for conflict concerning support for NBS with short-, medium-, or long-term effectiveness.

To achieve these objectives, we employed a PPGIS approach to collect and map citizens’ preferences for different types of NBS, categorized by their temporal effectiveness. In doing so, we expand the application of PPGIS to assess the emergence of potential land-use conflicts related to the temporal effectiveness of NBS and discuss their implication in the planning of NBS. This study is organized as follows: Section “Results” presents the main findings, which are further discussed in Section “Discussion”. Finally, Section “Methods” outlines the methodology employed for conducting the participatory survey, along with the spatial and aspatial analyses.

## Results

### PCA and cluster analysis

We selected the first five principal component from PCA that represented distinct preferences on the implementation of NBS: PC 1 - Opposition to NBS, PC 2 - Support for short-term NBS, PC 3 - NBS are not necessary, PC 4 - Support for medium-term NBS, PC 5 - Support for long-term NBS. Supplementary Table 1 presents the mean value and standard deviation of each item, as well as the Eigenvalue and Cronbach’s alpha for each component, confirming the validity and reliability of the identified dimensions.

These components were then used as input for the k-means cluster analysis to identify clusters of respondents with different temporal preferences for NBS. To avoid redundancy, the component “NBS are not necessary” was excluded from the clustering process, as its conceptual meaning overlaps with that of the component “Opposition to NBS”. The cluster centroids, representing the normalized mean values for each principal component, were analyzed to characterize the groups. These centroids illustrate how the clusters vary across the main principal components identified by the PCA (Table 1).

**Table 1:** Mean differences in factor scores by component and cluster group

Component	Cluster 1	Cluster 2	Cluster 3	C1 vs C2		C1 vs C3		C2 vs C3	
	Mean (SD)	Mean (SD)	Mean (SD)	*t*	*p*-value	*t*	*p*-value	*t*	*p*-value
Opposition to NBS	0.32 (0.77)	−0.95 (0.59)	0.75 (0.68)	−12.70	*p* < 0.001	−3.93	*p* < 0.001	−18.66	*p* < 0.001
Support for short-term NBS	0.14 (0.72)	0.61 (0.72)	−0.80 (0.97)	4.57	*p* < 0.001	7.47	*p* < 0.001	11.57	*p* < 0.001
Support for medium-term NBS	−0.53 (0.80)	0.78 (0.72)	−0.36 (0.90)	11.73	*p* < 0.001	−1.31	0.76	9.73	*p* < 0.001
Support for long-term NBS	−0.97 (0.88)	0.22 (0.68)	0.67 (0.65)	10.36	*p* < 0.001	−14.23	*p* < 0.001	−4.71	*p* < 0.001
Number of respondents	88	104	94						

Three clusters of respondents emerged from the analysis:

1. Respondents showing a clear preference for Short-Term NBS and limited support for other categories (hereinafter Cluster ST);
2. Respondents who generally support all types of NBS, with a preference for Medium-Term interventions (hereinafter Cluster MT);
3. Respondents with a strong preference for Long-Term NBS and limited support for other categories (hereinafter Cluster LT).

Pairwise t-tests were conducted to identify significant differences in the mean scores of the components between clusters, with Bonferroni correction applied to control for multiple comparisons. All components were statistically different between clusters, except for the “Support for medium-term NBS” component, where no significant differences emerged between Cluster ST and Cluster LT. This result is consistent with the characterization of these clusters, as their focus on short- and long-term NBS can excludes a clear distinction in support for medium-term solutions.

Within each cluster, we calculated the mean and standard deviation of each item and conducted pairwise comparisons using independent-sample t-tests (Supplementary Table 2). It is important to note that the component “Opposition to NBS” includes negatively worded items, meaning that lower scores indicate a lower degree of opposition (or greater support) for NBS.

Supplementary Table 3 show the distribution of the socio-demographic characteristics across the three clusters.

### Mapped points

A total of 1285 points were mapped in support of NBS development (Table 2). The western and eastern districts of Trento were merged due to the low number of points mapped in these areas, likely because these districts are already characterized by a high presence of natural areas. Overall, the category with the highest number of mapped points was long-term NBS, while the amount of support for the other two categories was similar. Cluster MT mapped nearly the same number of points for medium-term and long-term NBS, with fewer points mapped for short-term NBS. Cluster ST showed a preference for short-term NBS, and a nearly equal number of points for medium-term and long-term NBS. The main difference in mapped points was found in Cluster LT, which showed a strong preference for long-term NBS, mapping more than double the points compared to the other two categories. This cluster also showed a slight preference for medium-term compared to short-term NBS.

**Table 2:** Distribution of mapped points by NBS type, cluster and district

	East Trento	West Trento	Centro - Piedicastello	Gardolo	Mattarello	Oltrefersina	S. Giuseppe – S. Chiara	Total mapped points
Short-term NBS	11	17	187	12	6	56	78	367
Cluster 1 – Short-term	5 (45.5%)	8 (47.1%)	60 (32.1%)	4 (33.3%)	4 (66.7%)	20 (35.7%)	27 (34.6%)	128
Cluster 2 – Medium-term	3 (27.3%)	3 (17.6%)	64 (34.2%)	7 (58.3%)	1 (16.7%)	21 (37.5%)	34 (43.6%)	133
Cluster 3 – Long-term	3 (27.3%)	6 (35.3%)	63 (33.7%)	1 (8.3%)	1 (16.7%)	15 (26.8%)	17 (21.8%)	106
Medium-term NBS	8	25	162	21	11	80	72	379
Cluster 1 – Short-term	1 (12.5%)	5 (20%)	41 (25.3%)	2 (9.5%)	2 (18.2%)	16 (20%)	23 (31.9%)	90
Cluster 2 – Medium-term	6 (75%)	10 (40%)	65 (40.1%)	10 (47.6%)	7 (63.6%)	34 (42.5%)	26 (36.1%)	158
Cluster 3 – Long-term	1 (12.5%)	10 (40%)	56 (34.6%)	9 (42.9%)	2 (18.2%)	30 (37.5%)	23 (31.9%)	131
Long-term NBS	19	28	198	22	35	110	127	539
Cluster 1 – Short-term	4 (21.1%)	4 (14.3%)	37 (18.7%)	5 (22.7%)	4 (11.4%)	20 (18.2%)	24 (18.9%)	98
Cluster 2 – Medium-term	4 (21.1%)	8 (28.6%)	58 (29.3%)	8 (36.4%)	35 (40%)	110 (31.8%)	127 (29.1%)	164
Cluster 3 – Long-term	11 (57.9%)	16 (57.1%)	103 (52%)	9 (40.9%)	17 (48.6%)	55 (50%)	66 (52%)	277
Total mapped point	38	70	547	55	52	246	277	1285

### Distribution of benefit preferences

For each mapped point, respondents were asked to indicate the two main benefits they wished to obtain from the corresponding NBS (Table 3). A preference for benefits related to heatwave mitigation, air quality improvement, and aesthetic enhancement that emerged consistently across all three temporal categories of NBS.

**Table 3:** Distribution of benefit preferences by NBS categories and clusters

	Air quality improvement	Heatwave mitigation	Biodiversity increase	Reduction of urban noise	Stormwater management	Recreation opportunities	Social cohesion	Aesthetic enhancement	Food production	Total benefits by cluster
Short-term NBS	98	149	93	48	20	72	78	151	25	
Cluster 1 – Short-term	26 (26.5%)	46 (31.1%)	28 (30.1%)	18 (37.5%)	31 (43.1%)	30 (38.4%)	30 (38.4%)	51 (33.8%)	6 (24%)	248
Cluster 2 – Medium-term	51 (52%)	63 (41.9%)	38 (40.9%)	26 (54.2%)	22 (30.6%)	27 (34.6%)	27 (34.6%)	57 (37.7%)	14 (56%)	306
Cluster 3 – Long-term	21 (21.4%)	40 (27%)	27 (29%)	4 (8.3%)	0 (0%)	21 (26.9%)	21 (26.9%)	43 (28.5%)	5 (20%)	180
Medium-term NBS	121	155	90	45	21	87	69	146	24	
Cluster 1 – Short-term	34 (28.1%)	38 (24.7%)	15 (16.7%)	13 (28.9%)	2 (9.5%)	19 (21.8%)	25 (36.2%)	37 (25.3%)	3 (12.5%)	186
Cluster 2 – Medium-term	54 (44.6%)	60 (39%)	43 (47.8%)	21 (46.7%)	17 (81%)	40 (46%)	27 (39.1%)	64 (43.8%)	14 (58.3%)	340
Cluster 3 – Long-term	33 (27.7%)	57 (36.4%)	32 (35.6%)	11 (24.4%)	2 (9.5%)	28 (32.2%)	17 (24.6%)	45 (30.8%)	7 (29.2%)	232
Long-term NBS	204	259	152	63	17	108	75	160	40	
Cluster 1 – Short-term	40 (19.6%)	50 (19.3%)	23 (15.1%)	21 (33.3%)	2 (11.8%)	18 (16.7%)	16 (21.3%)	27 (16.9%)	3 (7.5%)	200
Cluster 2 – Medium-term	84 (41.2%)	80 (30.9%)	60 (39.5%)	21 (33.3%)	14 (82.4%)	27 (25%)	24 (32%)	59 (36.9%)	15 (37.5%)	384
Cluster 3 – Long-term	80 (39.2%)	129 (49.8%)	69 (45.4%)	21 (33.3%)	1 (5.9%)	63 (58.3%)	35 (46.7%)	74 (46.3%)	22 (55%)	494
Total benefits by NBS category	421	561	335	156	58	267	223	457	89	

Analyzing the preferences of individual clusters, Cluster MT prioritized benefits such as heatwave mitigation, air quality improvement, and aesthetic enhancement for both short-term and medium-term NBS, while biodiversity increase was also highlighted for long-term NBS. Cluster ST expressed priorities of heatwave mitigation and aesthetic enhancement for short-term NBS, adding air quality improvement for medium-term NBS, whereas for long-term NBS, air quality improvement and heatwave mitigation were preferred. Finally, Cluster LT showed a preference for heatwave mitigation and aesthetic enhancement for both short-term and medium-term NBS, with the addition of air quality improvement for long-term NBS.

### Directionality of preference and intensity of conflict for the support of NBS with different temporal effectiveness

**Fig. 1:**
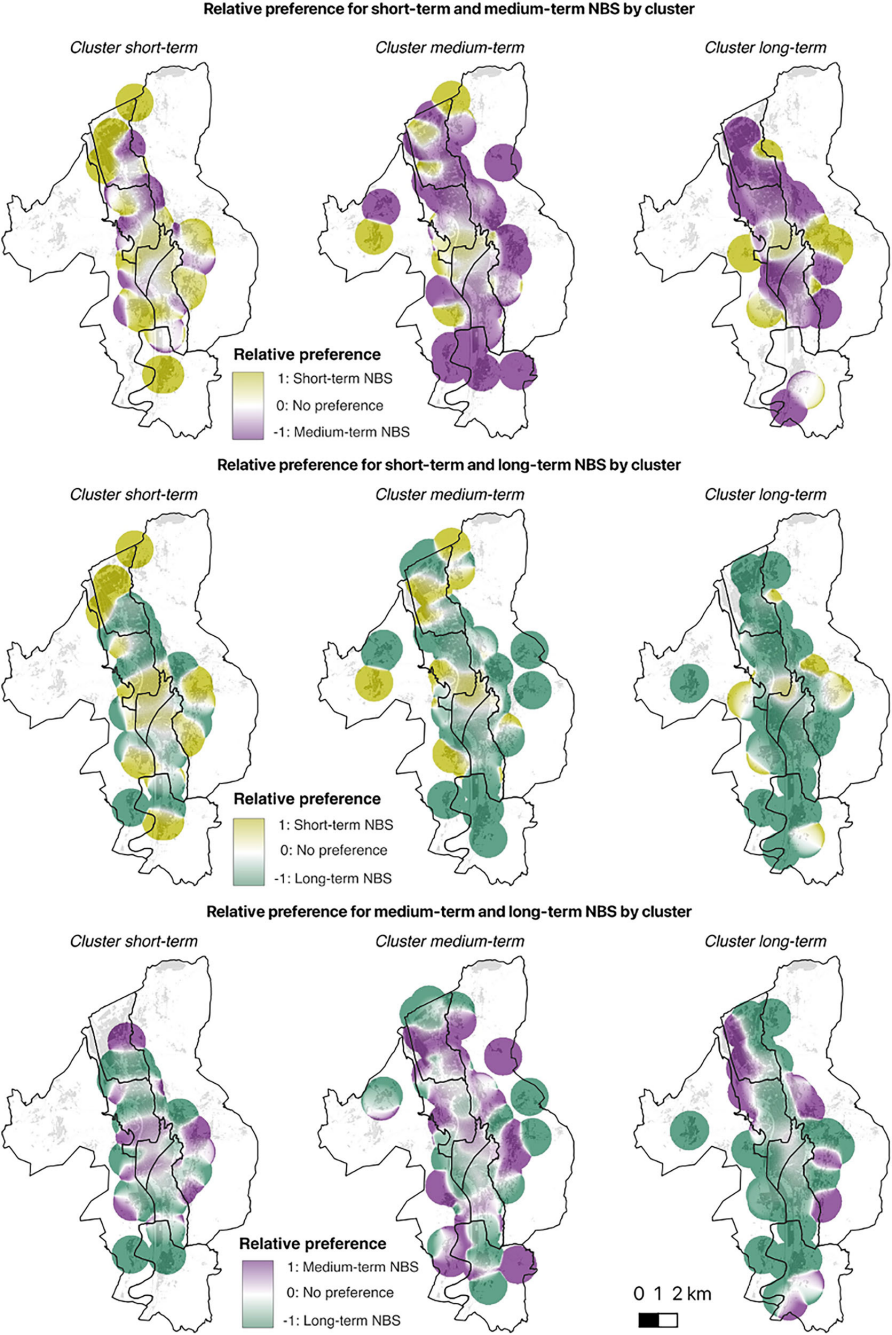
Relative preferences for the development of NBS with different temporal effectiveness by cluster.

For each pair of NBS categories, we identified the directionality of preference and intensity of conflict in the three clusters. Table 4 shows the mean pixel value for relative preference and conflict by location. The three analyses reveal substantial differences in the relative preferences expressed by the clusters, as shown in Fig. 1.

**Table 4:**
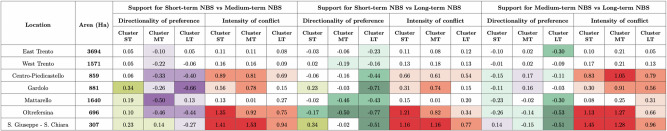
Mean pixel values for relative preference and conflict regarding supports for NBS with different temporal effectiveness by location

Directionality of preference – yellow cells indicate support for Short-terms NBS, purple cells indicate support for Medium-terms NBS, and green cells indicate support for Long-terms NBS

Intensity of conflicts – darker shades of red indicate higher levels of conflict.

The analysis of relative preferences shows that, in general, the clusters tend to favor the preferred NBS category when compared with other NBS, although some specific exceptions emerge for Clusters ST and MT. This highlights that preferences for the temporal effectiveness of NBS are strongly dependent on the local context. Cluster ST shows minor exceptions when short-term NBS are compared to long-term NBS. However, when short-term NBS are compared with medium-term NBS, Cluster ST demonstrates a clear preference for short-term NBS across all districts. Cluster MT presents a main exception in the central district of S. Giuseppe and Santa Chiara, both when medium-term NBS are compared with short-term NBS and when they are compared with long-term NBS. In contrast, Cluster LT shows a preference for long-term NBS in all comparisons. The analysis also shows how preferences are distributed when the preferred NBS category of a cluster is not included in the comparison. In these cases, both Cluster ST and Cluster MT tend to favor long-term NBS when compared with medium-term and short-term alternatives, respectively. Conversely, Cluster LT demonstrates a preference for medium-term NBS when compared with short-term NBS.

**Fig. 2:**
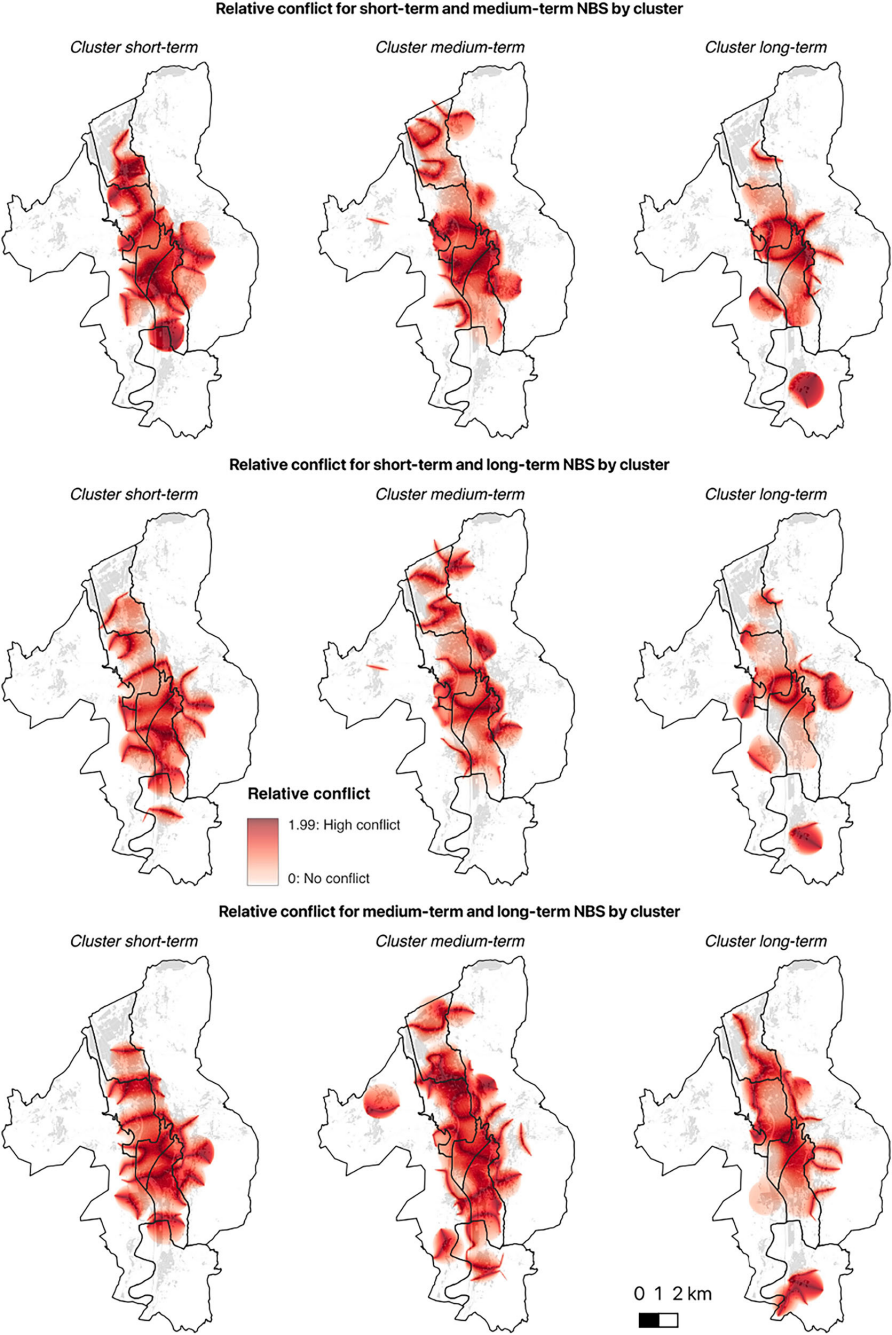
Relative conflict for the development of NBS with different temporal effectiveness by cluster.

The intensity of the conflict varies depending on the category of NBS compared, the clusters considered, and the geographic areas (Fig. 2). In particular, central neighborhoods consistently show higher levels of conflict across all cases analyzed. In these areas, conflict arises even when the preferred category of NBS is not included in the comparison. In general, in cases of conflict, the direction of preference tends to remain oriented towards the reference NBS for all clusters. Exceptions are observed with Cluster MT, which showed a high level of conflict in the S. Giuseppe and Santa Chiara neighborhood. In this area, Cluster MT expressed a relative preference for short-term NBS when compared with medium-term NBS, and a preference for long-term NBS when compared with medium-term NBS. Furthermore, Cluster ST exhibits a preference for long-term NBS in the Oltrefersina neighborhood.

## Discussion

We present a PPGIS study that spatially assesses relative preferences and potential conflicts regarding the implementation of NBS with different temporal effectiveness, i.e., NBS that provide the full range of benefits in the short, medium, or long term in different intensity. We explored public preferences by dividing the population into three clusters that grouped together respondents with similar level of support for a specific NBS temporal category. The main contribution of our study is the introduction of the concept of NBS temporal effectiveness and the spatial assessment of its role in shaping public preferences for NBS, an aspect previously overlooked in the literature^20,21^. The classification of NBS into three temporal categories and the introduction of the trade-off between the immediacy and magnitude of benefits provide a theoretical framework useful for simplifying and structuring the analysis of temporal preferences. This prompted respondents to reflect on the extent to which they would be willing to sacrifice more substantial benefits for greater rapidity, or conversely, to forgo rapidity in favor of more impactful benefits. Additionally, the use of PPGIS required them to apply this reasoning specifically to each area where they would support the implementation of an NBS, taking into account the contextual needs. Our results indicate support for all temporal categories of NBS, with a higher preference for those with long-term effectiveness. The intensity and direction of conflict vary depending on the pairs of NBS temporal categories being compared, respondent groups, and urban contexts.

Previous PPGIS studies had shown that preferences for NBS vary according to factors such as landscape attributes^42,54^, the level of vegetation^55,56^, sociodemographic characteristics such as gender^57,58^, ethnicity^57^, age^59^, education^60^, proximity to home^55,61^, and perceived benefits and values^62^. Our study provides a significant contribution to the PPGIS literature by demonstrating for the first time, that temporal effectiveness is also a key factor influencing preferences for NBS. Responses to motivational questions, along with the number of mapped points, indicate a general support of all temporal categories, aligning with previous studies that found that people generally support green spaces^42^, and reveal a stronger preference for long-term NBS. This finding constitutes a significant contribution to the NBS literature, as existing studies have often identified the temporal lag in benefit production as a barrier to their implementation^33,34^. In contrast, our results indicate that citizens not only support but often prefer interventions requiring over 10 years to deliver the full range of benefits, as long as these benefits are more substantial than those of more immediate NBS. This highlights the importance of clearly communicating this aspect when planning for long-term NBS to reduce initial skepticism toward interventions whose positive effects are not immediately visible. Furthermore, it could lead to increased public support for NBS when compared to gray infrastructure, which is often favored due to its capacity to provide immediate results^9^.

The application of the methodology proposed by Brown and Raymond^63^, and Lechner et al.^52^, enabled the analysis of the directionality and intensity of conflict, between and within population groups with different attitudes. This allowed for a more detailed understanding of the distribution of preferences, identifying areas, clusters, and types of interventions most subject to divergences of opinion^52,64^. Respondents belonging to a specific cluster, while generally showing a preference for a particular category of NBS, in some areas revealed stronger preferences for other categories. This is consistent with previous conflict analyses using PPGIS^52^. This flexibility in preferences was observed particularly in the short- and medium-term. In contrast, the long-term cluster demonstrated a more consistent preference for long-term NBS across all areas. This tendency may arise from an aversion to low-impact public interventions, which are perceived as quick fixes aimed at rapidly gaining public support but potentially requiring additional investment in the future. This is confirmed by the responses from this cluster to the motivational questions (see Supplementary Table 2).

Despite the importance of territorial context, our analysis shows that it is not always possible to identify an unanimously preferred category of NBS for a specific area across all clusters. This is consistent with previous PPGIS studies^65^ that showed that patterns linking intervention areas to specific land uses occasionally emerge, and that preferences for NBS are not solely linked to objective territorial characteristics but are also influenced by personal factors and priorities that may be difficult to capture^45,66^. For example, there seems to be a tendency to prefer short-term NBS in densely populated central urban areas, where there is a pressing need to quickly improve quality of life. Conversely, these interventions tend to lack support in peripheral areas, such as low-density residential neighborhoods and agricultural zones. However, these patterns are not consistent across all clusters, highlighting the complexity of the issue and the need to implement multiple solutions in certain areas, as observed in previous studies^67^. This emphasizes the need for participatory approaches, and future studies could delve deeper into the personal motivations behind these preferences. Future studies could also conduct analyses to understand how temporal preferences vary in relation to more specific urban attributes, such as land use, proximity to home, availability of green spaces, neighborhood density, or the socio-economic characteristics of the area.

Conflicting preferences were also identified within the same clusters, leading to the identification of areas of potential conflict. In these areas, it is important to further investigate the reasons behind the disagreements and find acceptable solutions, for example, by engaging local stakeholders to identify solutions that balance different preferences for NBS^42,65,68^. Our results show that preference conflicts are more intense in central neighborhoods, characterized by higher population density and limited green spaces. These are the most frequented areas of the city and, as a result, are subject to the opinions of a larger number of people. These areas received the highest number of mapped points, consistent with findings from previous PPGIS studies^69^. This concentration can be partially explained by the phenomenon of spatial discounting, i.e., people tend to favor locations that are easily accessible and close to home, as highlighted in prior research^65,67^.

Our study presents a new perspective on NBS planning by focusing on the consideration of their temporal effectiveness and the preferences that people have regarding it. We found that citizens have specific preferences regarding the timing and impact of the benefits associated with NBS, and that such preferences are place-specific and vary across different population groups. Furthermore, we discovered that differing temporal preferences can, in some cases, lead to potential conflicts. Taking these factors into account can enable the adoption of more inclusive and targeted strategies, enhancing both the effectiveness and the acceptability of NBS interventions.

Previous studies have shown that the temporal lag of NBS can act as a barrier to their implementation^6,34^. Adopting long-term NBS in contexts where the community demands immediate solutions, or vice versa, risks failing to meet citizens’ expectations regarding the capacity of NBS to address environmental and social challenges. This challenge can be overcome by planning NBS interventions that account for the temporal needs of local communities, increasing their acceptability and improving citizens’ perceptions of their effectiveness. Moreover, technical solutions are often perceived as more favorable than NBS because they provide immediate results^9^. Our study shows that people can provide greater support for long-term NBS when they demonstrate to provide greater overall benefits compared to immediate solutions. Furthermore, the analysis of preference directionality provides an additional tool to understand whether, and to what extent, the planned NBS is acceptable to the local community in a specific area^63^. This approach facilitates aligning planning choices with the needs of the communities, fostering collaboration among different social groups^64^; and contributing to greater acceptance of the proposed solutions.

This study has some limitations. First, we acknowledges that the classification of NBS into three temporal categories (short, medium, and long term) represents a simplification. We are aware that the different ES provided by the same NBS may follow different temporal dynamics and do not always reflect a clear trade-off between the immediacy and magnitude of benefits and that NBS and ES temporal patterns are not always linear. However, since this study represents a first attempt to understand the role of temporality in public preferences for NBS, we believe that this classification can serve as a reasonable starting point for capturing preliminary insights into the issue of NBS temporality, even though it does not fully reflect the complexity of ecosystem dynamics. Future studies could integrate preference analysis with simulation models of NBS temporal dynamics to improve the precision of the results and provide a more comprehensive understanding of how citizens’ preferences align with the actual temporal dynamics of NBS. In addition, we recommend that future studies on NBS undertake dynamic analyses of NBS effectiveness over time, as this crucial aspect remains largely underestimated in the literature.

A further limitation of our study is the overrepresentation of younger generations, which may have influenced the results. Moreover, the use of convenience and voluntary sampling might have affected the representativeness of the sample and favored the inclusion of individuals more inclined to prefer a specific temporal typology of NBS. Issues related to sample size and representativeness are common in PPGIS studies^46,70,71^, and previous PPGIS studies have shown that sociodemographic biases linked to different sampling techniques have a limited influence on mapped data^67,69^. To improve the validity and generalizability of findings, future research should consider adopting more representative sampling methods, such as random sampling, and aim for a larger and more balanced sample^72^.

## Methods

### Study area

**Fig. 3:**
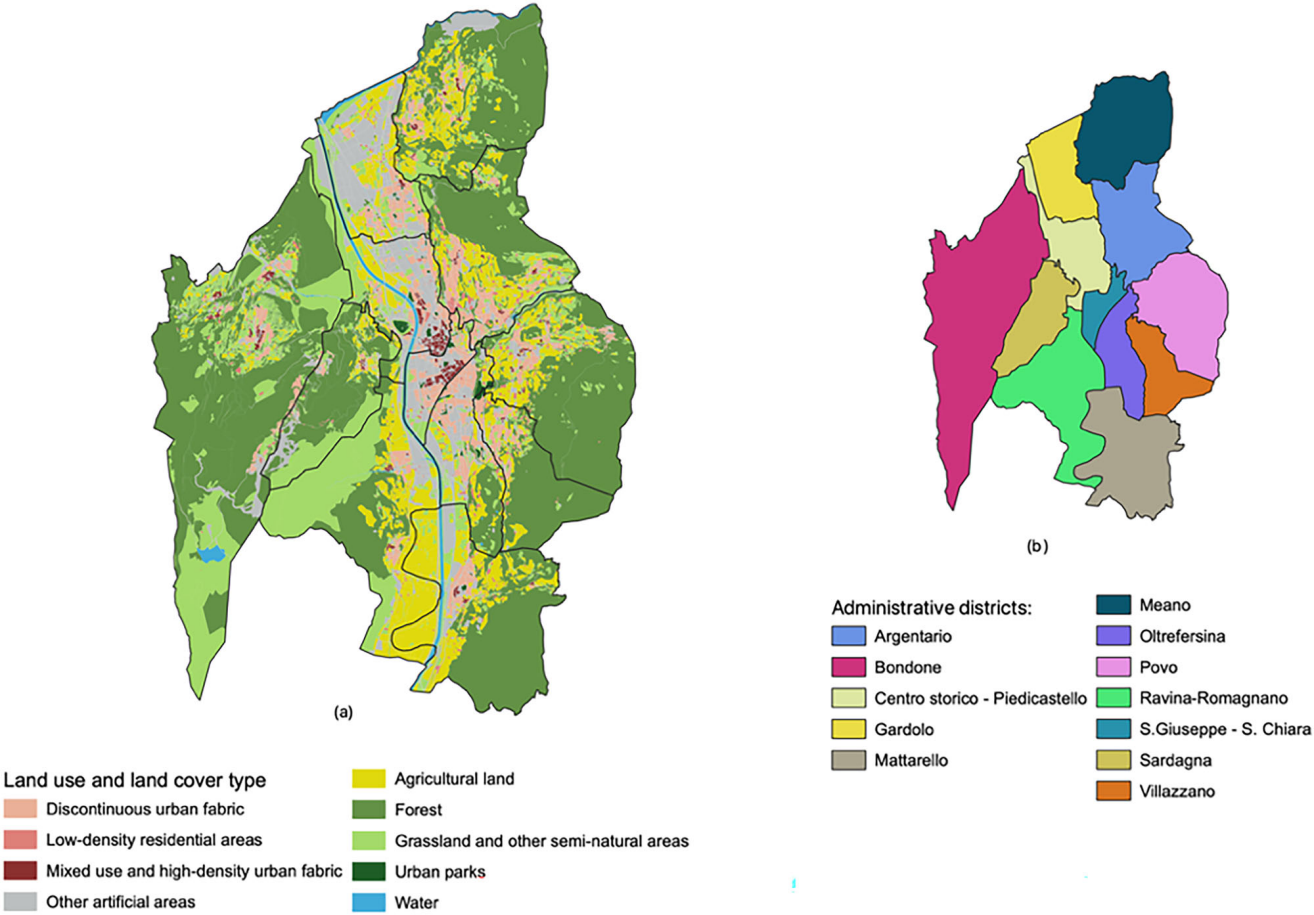
Land use and administrative subdivisions in the Municipality of Trento. **a** Distribution of land use and land cover types. **b** Administrative districts of Trento.

Trento is an Alpine city of ~120,000 inhabitants, located in the Eastern Italian Alps. The administrative area of the city spans over 158 km², of which 72 km² are covered by forests (Fig. 3a). Around 70% of the population lives on the valley floor, where most of the industrial and commercial areas and infrastructure are also concentrated. Agricultural areas, mainly vineyards and apple orchards, occupy the non-urbanized patches on the valley floor and the sunny hillsides. The rest of the population resides in small villages scattered across the surrounding hills and mountains.

The city of Trento is divided into 12 administrative districts (Fig. 3b), each with specific local responsibilities. Each district represents a territorial area with individual characteristics, encompassing neighborhoods that vary in terms of population density, economic activities, and distribution of, and access to, different types of green and blue spaces^73,74^. In the urbanized area of Trento, private gardens cover 4.6% of the municipal area, while public green spaces occupy 2%^52,75^. A recent survey conducted by the Municipality of Trento shows that one in three residents visits a park daily, and almost 70% of residents are frequent users of green areas, highlighting their importance to the community^75^. Currently, the Municipality of Trento is actively engaged in several projects aimed at increasing green spaces, promoting biodiversity, and developing green infrastructure.

### Respondent sampling

The target population for our study consisted of Trento residents aged 18 and above. To reach city residents, we used a convenience and voluntary sampling approach, where participants were recruited through survey invitations shared via email, and social media platforms (e.g., Facebook and LinkedIn), an in person using flyers distributed across the city^76,77^. Those who consented to participate completed the survey individually. A total of 286 respondents responded to the survey. Supplementary Table 4 compares the sociodemographic characteristics of the Trento population (Census data) with those of our sample. The gender distribution among respondents—46.2% female (*n* = 132) and 51.8% male (*n* = 148)—aligns reasonably well with that of the population (51.9% female and 48.1% male). Younger age groups are overrepresented in our sample, particularly those aged 18–34, who make up 52.1% of our respondents compared to 23.6% of the population. Conversely, older age groups are underrepresented, with individuals aged 55 and above comprising only 12.6% of our sample compared to 46.2% in the overall population. Underrepresentation of certain socio-demographic groups (e.g. the elderly) in PPGIS studies have been widely acknowledged in literature and therefore sampling biases should be considered when interpreting the findings^72,78,79^.

### PPGIS survey design

The data for this study was collected between July 12, 2024, and September 1, 2024, using an online survey format via the participatory mapping platform Maptionnaire. The survey collected spatial and aspatial data on respondents’ support or opposition to developing NBS with different temporal effectiveness in Trento (for full survey, see Supplementary Note 1). Initially, participants were provided with detailed information about the research project and gave informed consent to participate, with the option to withdraw from the survey at any point. Respondents had the option to complete the survey in either Italian or English.

The survey included multiple-choice questions, Likert-scale questions, and mapping tasks. In the first section, respondents were asked to identify their residence on a 400 × 400 m^2^ grid to ensure privacy. In the second section, we described and provided general examples of the three NBS temporal categories based on our proposed classification of NBS temporal effectiveness. These categories were designed to require respondents to consider a trade-off between the rapidity at which the benefits could be realized and their magnitude, acknowledging that an NBS delivering faster results might provide smaller benefits, and vice versa (Table 5).

**Table 5:** Description of the NBS temporal categories used in the survey

Label	NBS temporal category	Description
Short-term NBS	NBS with short-term effectiveness	Provide limited environmental and social benefits that appear within a year of their creation. The vegetated components of the NBS can include grassy meadows and annual plants.
Medium-term NBS	NBS with medium-term effectiveness	Offer greater environmental and social benefits compared to short-term NBS. However, these benefits only emerge 3–5 years after the area’s creation. The vegetated components of these areas can include a combination of annual plants and fast-growing trees.
Long-term NBS	NBS with long-term effectiveness	Provide broader environmental and social benefits compared to medium-term NBS, offering significant and lasting impact. However, these benefits only emerge within 6–10 years of the NBS implementation. The vegetated component of this NBS can include parks with trees and perennial plants or tree-lined avenues.

For each category of NBS, respondents were asked to map where they would support their implementation. They were allowed to map as many points as they wished, with no restrictions on the minimum or maximum number of markers. For each mapped location, respondents were asked to select two environmental or social co-benefits from a list of nine, indicating the ones they aimed to achieve with that specific NBS. Similarly, in the third section, we asked respondents to map where they would oppose the development of short-, medium-, or long-term NBS. At the end of the second and third sections, respondents answered 1–5 Likert-scale questions about their motivations for supporting or opposing the three typologies of NBS. These motivation questions were based on insights derived from prior interviews with Trento stakeholders, aimed at identifying the reasons for supporting or opposing the implementation of NBS with short- and long-term effectiveness. These responses were analyzed and coded using Maxqda^80^ software and included in the survey. Finally, in the last section, we collected socio-demographic data.

### Clustering respondent in groups

We conducted a Principal Component Analysis (PCA) to group the motivational questions regarding support and opposition to NBS with different temporal effectiveness. PCA is a statistical technique that reduces a dataset of multiple variables to a few principal components—linear combinations of the original variables that capture the maximum variance—providing a simplified approximation of the original data^81^. The findings from the PCA were then used to inform the clustering of respondents^82^. The analyses were conducted using R software. The PCA was performed on a dataset that included the responses to the 27 motivational questions from the survey. This analysis was conducted to reduce the dimensionality of the motivational items. We employed an oblique rotation method to allow for correlation between components^82^, thereby capturing the complexity of social phenomena more accurately. For each component, we calculated the factor scores and finally selected the first five components, as they were the ones with eigenvalues greater than 1, that explained 60% of the total variance. The interpretation of each component was informed by the factor loadings of the items, which reflect the strength of their association with the respective component. The reliability of all components was assessed using Cronbach’s alpha, with α ≥ 0.7 considered sufficient, indicating the internal consistency of the grouped motivational items^83^. These measures help address common critiques of PCA- such as forced orthogonality, weak interpretability, and insufficient verification of component consistency^84^- by enhancing the interpretability and stability of the derived components. We then used these components as input for a K-means cluster analysis, aiming to divide respondents into groups that reflect the attitudes toward supporting NBS with different levels of temporal effectiveness. Cluster analysis, however, has some inherent limitations, including its reliance on specific variables and the fact that it will generate clusters even when no meaningful structure exists in the data^84^. To mitigate these issues, we included in the K-means analysis, only components with sufficient reliability scores, and conducted pairwise t-tests to assess whether the mean PCA scores differed significantly between each pair of clusters, followed by Bonferroni corrections to adjust for multiple comparisons^85^. The adjusted *p*-values were computed to determine the significance of the differences between clusters, setting an alpha level of 0.05. This approach helps validate the distinctiveness of each cluster and partially addresses concerns regarding the stability and interpretability of the resulting groups.

We then analyzed the distribution of prioritized benefits across clusters and categories of NBS to identify patterns linking cluster membership with the prioritized benefits, as well as the benefits expected from each category of NBS.

### Spatial analysis

For each cluster, we assessed the directionality of preference and the intensity of conflict for each pair of NBS typologies (short-term NBS vs. medium-term NBS; short-term NBS vs. long-term NBS; medium-term NBS vs. long-term NBS). This analysis was performed following the methodology proposed by Brown and Raymond^63^ and Lechner et al.^52^, using QGIS 3.22 software. As a preliminary step, we cleaned the dataset by removing duplicate points mapped in the same areas by the same respondent. Using Kernel Density Estimation (KDE), we created nine density maps, one for each pair of NBS typologies and clusters. The KDE search radius was determined through an adaptation of Silverman’s rule-of-thumb bandwidth estimation formula^86^ and a heuristic approach by testing different size buffers for maximum data inclusion and appropriate level of detail, while the grid size was determined using Eq. (1):

1 $$\,p\le \frac{\overline{{h}_{ij}}}{2}$$

where ${h}_{{ij}}$ represents the mean nearest neighbour distance between point pairs^87^. We estimated these parameters for each of the nine datasets, and then used their average value for all KDE estimates. The final search radius was 1000 m, and the grid size was set to 25 m. We kept a smaller grid size to improve the map resolution.

For each cluster, we then assessed the directionality of preference for each pair of NBS temporal category using Eq. (2). For example, to assess the directionality of preference between short-term and medium-term NBS, the pixel values were calculated using Eq. (2) as follows:

2 $$\,{Relative\; preference\; for\; ST\; and\; MT\; NBS}=\frac{{Support\; ST}-{Support\; MT}}{\max \left(\left|SupportST,SupportMT\right|\right)}$$

where “Support ST” and “Support MT” are the pixel values derived from the density of PPGIS points in each grid cell using KDE for the support of short-term NBS and medium-term NBS respectively. Values were rescaled to range from 0 to 1 to indicate the strength of support for each NBS category, while negative values (ranging from −1 to 0) indicated stronger support for the compared NBS category. The rescaling was based on the maximum pixel value recorded. This approach was then consistently applied to all pairs of NBS typologies within each cluster.

Afterward, we estimated the intensity of conflict following Eq. (3) proposed by Brown and Raymond^63^ and Lechner et al.^52^:

3 $$\,{Relative\; conflict\; for\; ST\; and\; MT\; NBS}=\frac{\min \left({Support\; ST},{Support\; MT}\right)\times \,2}{\max \left(\left|SupportST,SupportMT\right|\right)}$$

The relative conflict analysis involved rescaling the maximum absolute pixel value, irrespective of sign, calculated as the difference between NBS temporal categories. Low relative conflict values indicate a general consensus on the preferred NBS category in that pixel area, whereas high values indicate opposing preferences. The same rescaling value was used as in the relative preference analysis, allowing for direct comparison between relative preference and conflict. For example, a high value of relative preference (e.g., indicating preference for short-term NBS) combined with a high conflict value suggests that, despite a preference for short-term NBS, there is also significant support for medium-term NBS in that area. The analysis was performed for each pair of NBS typologies within each cluster. Finally, we estimated the mean value of directionality and conflict in each district of Trento.

## Supplementary information

Supplementary material

## Data Availability

The datasets generated and analyzed during the current study are not publicly available due to privacy restrictions but are available from the corresponding author on reasonable request.

## Code availability

The underlying code for this study is not publicly available but may be made available to qualified researchers on reasonable request from the corresponding author.
