# Supplementary material for: Exploring citizens’ preferences for the temporal effectiveness of urban nature-based solutions through participatory GIS
Source: NPJ Urban Sustain. 2025 Jul 1;5(1):44. doi: 10.1038/s42949-025-00229-5 (PMC12213601; doi:10.1038/s42949-025-00229-5)
Supplement: Supplementary file 1 — Supplementary material [file 42949_2025_229_MOESM1_ESM.pdf]

Supplementary material

Supplementary Table 1: Results of the Principal Component Analysis for Support and Opposition to NBS

	Mean	S.D.	St. factor loading	Eigenvalue	% of variance	Cumulative %	Cronbach's α
**PC 1 - Opposition to NBS**				8.637	0.22	0.22	0.93
Short-term green areas require additional investments to address environmental and/or social problems	2.87	1.360	0.65				
Short-term green areas do not provide significant environmental and/or social improvements	2.38	1.198	0.53				
Short-term green areas do not take into account the needs of future generations	2.62	1.278	0.71				
Short-term green areas are measures aimed at temporarily satisfying public opinion	2.72	1.233	0.64				
Medium-term green areas attract less public support because they do not yield immediate results	2.72	1.205	0.80				
Medium-term green areas do not take into account the needs of future generations	2.16	0.971	0.58				
Medium-term green areas do not guarantee the achievement of the expected benefits	2.35	1.031	0.66				
Medium-term green areas do not provide significant environmental and/or social improvements	2.10	0.944	0.52				
Long-term green areas do not respond quickly to current environmental and/or social needs	2.68	1.328	0.82				
Long-term green areas attract less public support because they do not yield immediate results	2.69	1.410	0.93				
Long-term green areas do not guarantee the achievement of the expected benefits	2.20	1.202	0.72				
**PC 2 - Support short-term NBS**				2.720	0.10	0.32	0.76
Short-term green areas help raise awareness about the importance of these interventions for urban sustainability	4.00	0.869	0.77				
Short-term green areas provide a quick response to current environmental and/or social needs	3.99	0.898	0.81				
Short-term green areas encourage the development of additional green interventions in the future	3.94	0.982	0.64				
Short-term green areas are a reliable source of environmental and/or social benefits	3.82	0.878	0.74				
**PC 3 - NBS are not necessary**				2.090	0.10	0.42	0.72
Short-term green areas are not necessary	1.74	0.836	0.74				
Medium-term green areas are not necessary	1.57	0.768	0.79				
Long-term green areas are not necessary	1.34	0.796	0.67				
**PC 4 - Support medium-term NBS**				1.555	0.09	0.51	0.72
Medium-term green areas help raise awareness about the importance of these interventions for urban sustainability	4.02	0.805	0.59				
Medium-term green areas represent a fair compromise between the extent of the benefits offered and the time it takes for them to manifest	4.10	0.797	0.66				
Medium-term green areas encourage the development of additional green interventions in the future	3.96	0.826	0.72				
Medium-term green areas provide a quick response to current environmental and/or social needs	3.76	0.879	0.70				
**PC 5 - Support long-term NBS**				1.094	0.08	0.60	0.71
Long-term green areas help raise awareness about the importance of these interventions for urban sustainability	4.23	0.839	0.59				
Long-term green areas reduce the need for additional investments to address environmental and/or social issues	4.07	0.892	0.66				
Long-term green areas encourage the development of additional green interventions in the future	4.05	0.907	0.49				
Long-term green areas offer significant environmental and/or social improvements	4.44	0.697	0.59				
Long-term green areas address the needs of future generations	4.48	0.719	0.53				

Supplementary Table 2: Mean differences in scores by component items and cluster group

Items	Cluster 1 Mean (SD)	Cluster 2 Mean (SD)	Cluster 3 Mean (SD)	C1 vs C2		C1 vs C3		C2 vs C3	
				t	p-value	t	p-value	t	p-value
**Opposition to NBS**									
OST - Need for additional investments	2.93 (1.07)	1.82 (1.04)	3.99 (0.94)	-7.30	$p < 0.001$	-7.08	$p < 0.001$	-15.32	$p < 0.001$
OST - Not significant improvements	2.32 (0.93)	1.49 (0.70)	3.41 (1.04)	-7.04	$p < 0.001$	-7.48	$p < 0.001$	-15.42	$p < 0.001$
OST - Future generations not considered	2.66 (0.98)	1.64 (0.91)	3.67 (1.00)	-7.42	$p < 0.001$	-6.88	$p < 0.001$	-14.91	$p < 0.001$
OST - Temporary public satisfaction	2.78 (0.95)	1.80 (0.92)	3.69 (0.97)	-7.25	$p < 0.001$	-6.35	$p < 0.001$	-14.01	$p < 0.001$
OMT - No public support	3.17 (1.03)	1.70 (0.80)	3.43 (0.94)	-11.11	$p < 0.001$	-1.74	0.083	-13.90	$p < 0.001$
OMT - Future generations not considered	2.27 (0.81)	1.49 (0.71)	2.80 (0.89)	-7.12	$p < 0.001$	-4.16	$p < 0.001$	-11.50	$p < 0.001$
OMT - Uncertainty in benefits provision	2.53 (0.86)	1.62 (0.70)	2.99 (0.99)	-8.17	$p < 0.001$	-3.31	$p < 0.001$	-11.36	$p < 0.001$
OMT - Not significant improvements	2.31 (0.85)	1.43 (0.59)	2.65 (0.91)	-8.39	$p < 0.001$	-2.61	0.010	-11.26	$p < 0.001$
OLM - Slow response	3.26 (1.21)	1.58 (0.83)	3.35 (1.06)	-11.38	$p < 0.001$	-0.53	0.595	-13.13	$p < 0.001$
OLT - No public support	3.32 (1.26)	1.53 (0.81)	3.39 (1.22)	-11.84	$p < 0.001$	-0.41	0.683	-12.77	$p < 0.001$
OLT - Uncertainty in benefits provision	2.49 (1.09)	1.48 (0.72)	2.72 (1.34)	-7.63	$p < 0.001$	-1.29	0.199	-8.23	$p < 0.001$
**Support for short-term NBS**									
SST - Increase awareness on NBS	3.98 (0.80)	4.48 (0.62)	3.50 (0.88)	4.89	$p < 0.001$	3.82	$p < 0.001$	9.14	$p < 0.001$
SST - Quick response	4.09 (0.80)	4.34 (0.69)	3.50 (0.98)	2.29	0.023	4.44	$p < 0.001$	6.99	$p < 0.001$
SST - Future development	3.98 (0.86)	4.46 (0.62)	3.32 (1.07)	4.52	$p < 0.001$	4.56	$p < 0.001$	9.29	$p < 0.001$
SST - Certanity in benefit provision	3.86 (0.76)	4.27 (0.73)	3.29 (0.85)	3.77	$p < 0.001$	4.81	$p < 0.001$	8.76	$p < 0.001$
**NBS are not necessary**									
OST - Not necessary	1.83 (0.90)	1.31 (0.54)	2.13 (0.83)	-4.95	$p < 0.001$	-2.32	0.021	-8.29	$p < 0.001$
OMT - Not necessary	1.83 (0.90)	1.25 (0.52)	1.68 (0.75)	-5.57	$p < 0.001$	1.21	0.227	-4.74	$p < 0.001$
OLT - Not necessary	1.61 (0.95)	1.17 (0.55)	1.28 (0.81)	-4.00	$p < 0.001$	2.58	0.017	-1.06	0.289
**Support for medium-term NBS**									
SMT - Increase awareness on NBS	3.81 (0.69)	4.57 (0.55)	3.63 (0.82)	8.46	$p < 0.001$	1.59	0.113	9.56	$p < 0.001$
SMT - Fair compromise	3.83 (0.76)	4.46 (0.64)	3.97 (0.85)	6.26	$p < 0.001$	-1.16	0.249	4.65	$p < 0.001$
SMT - Future development	3.55 (0.83)	4.44 (0.59)	3.82 (0.79)	8.74	$p < 0.001$	-2.28	$p < 0.001$	6.34	$p < 0.001$
SMT - Quick response	3.43 (0.81)	4.30 (0.70)	3.47 (0.84)	7.96	$p < 0.001$	-0.30	0.768	7.61	$p < 0.001$
**Support for long-term NBS**									
SLT - Increase awareness on NBS	3.56 (0.88)	4.59 (0.58)	4.47 (0.65)	9.66	$p < 0.001$	-7.96	$p < 0.001$	1.35	0.180
SLT - Reduce future investment needs	3.34 (0.91)	4.43 (0.69)	4.35 (0.63)	9.43	$p < 0.001$	-8.74	$p < 0.001$	0.86	0.390
SLT - Future development	3.31 (0.84)	4.54 (0.64)	4.19 (0.78)	11.57	$p < 0.001$	-7.39	$p < 0.001$	3.44	$p < 0.001$
SLT - Significant improvements	4.11 (0.82)	4.63 (0.56)	4.55 (0.60)	5.09	$p < 0.001$	-4.14	$p < 0.001$	0.87	0.384
SLT - Future generations considered	4.09 (0.84)	4.68 (0.51)	4.61 (0.66)	6.01	$p < 0.001$	-4.62	$p < 0.001$	0.92	0.360

Supplementary Table 3: Distribution of sociodemographic characteristics by cluster of respondents

	**CL1 - Short**	**CL2 - Medium**	**CL3 - Long**
**Gender**			
Female	28 (21%)	57 (43%)	47 (36%)
Male	58 (39%)	44 (30%)	46 (31%)
Other	2 (33%)	3 (50%)	1 (17%)
**Age**			
18-24	10 (21%)	19 (40%)	18 (38%)
25-34	31 (28%)	32 (36%)	39 (36%)
35-44	16 (49%)	21 (33%)	21 (19%)
45-54	21 (32%)	14 (50%)	8 (18%)
55-64	7 (32%)	11 (50%)	4 (29%)
65+	3 (21%)	7 (50%)	4 (29%)
**Education**			
Primary school	-	1 (33%)	2 (67%)
Middle school	7 (58%)	-	5 (42%)
High school	22 (25%)	37 (43%)	28 (32%)
B.Sc or M.Sc degree	43 (29%)	55 (37%)	50 (34%)
Doctoral degree	16 (44%)	11 (31%)	9 (25%)
**Job**			
Employed	61 (35%)	62 (35%)	52 (30%)
Unemployed	7 (50%)	4 (29%)	3 (21%)
Student	14 (19%)	30 (41%)	29 (40%)
Stay at home	2 (50%)	-	2 (50%)
Retired	3 (20%)	7 (47%)	5 (33%)
In other conditions	1 (20%)	1 (20%)	(60%)

Supplementary Table 4: Sociodemographic comparison of the survey respondents and the population in Trento in 2019 and 2024

	**Survey (n = 286)**	**Trento (n = 100 197)**
**Gender**		
Female	132 (46.2%)	51 980 (51.9%)
Male	148 (51.8%)	48 217 (48.1%)
Other	6 (2.1%)	
**Age**		
18-24	47 (16.4%)	8 678 (8.7%)
25-34	102 (35.7%)	14 222 (14.2%)
35-44	58 (20.3%)	14 225 (14.2%)
45-54	43 (15.0%)	16 757 (16.7%)
55-64	22 (7.7%)	17 656 (17.6%)
65+	14 (4.9%)	28 659 (28.6%)
**Education**		
Primary school	3 (1.1%)	12 815
Middle school	12 (4.2%)	26 555
High school	87 (30.4%)	43 401
Bachelor's or Master's degree	148 (51.6%)	23 820
Doctoral degree	36 (12.6%)	1 497
**Job**		
Employed	175 (61.2%)	53 977
Unemployed	14 (4.9%)	5 093
Student	73 (25.5%)	9 173
Stay at home	4 (1.4%)	6 883
Retired	15 (5.2%)	23 587
In other conditions	5 (1.8%)	5 561
**Household**	2.516	

Data on gender and age refer to the 2024 population over 18 years old. Education data refer to the 2019 population over 9 years old. Job data refer to the 2019 population over 15 years old.

Supplementary note 1

Survey structure

1. **Domicile - where do you live?**

Mark your home by clicking the "my home" button and selecting the corresponding cell. If you have more than one home, map your primary residence. Your residence will not be shared with anyone, and it will not be possible to identify the exact location. All responses will be treated anonymously.

Make sure to zoom in enough to place the marker accurately.

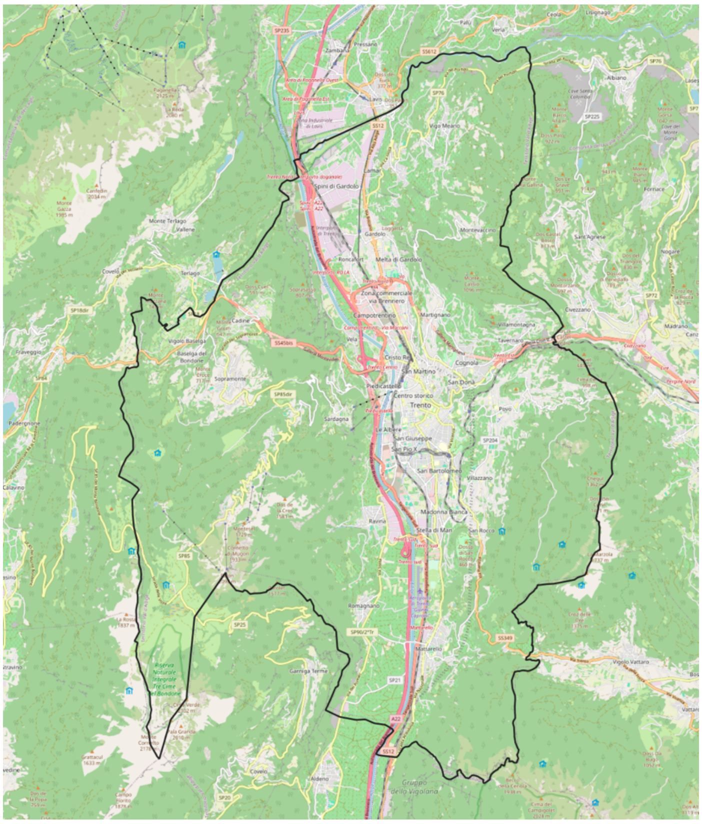

Key terms in the survey

With "green areas[1]" we refer to urban areas with vegetation, such as parks, urban woods, or tree-lined boulevards. These spaces can offer various environmental and social benefits. Environmental benefits include temperature reduction, air quality enhancement, stormwater management, carbon

[1] In this questionnaire, the term "green areas" has been used as a proxy for NBS to facilitate participants' understanding. The definition and examples provided within the survey are consistent with the established definition of NBS.

sequestration, and increased biodiversity. Social benefits include providing areas for relaxation, recreational activities, and improving the aesthetic quality of the landscape.

In the following sections, we will ask you to map where you would support or oppose the creation of three different types of green areas, which vary based on the quality and extent of the benefits offered and the time required for these benefits to materialize.

---

1. **Map where you would like to see new green areas created in Trento!**

Click the “short term”, “medium term”, and “long term” buttons below and place them on the map to indicate where you would like to see new green areas that provide benefits in the short, medium, and long term. You can place as many points as you like for each type of green area.

Make sure to zoom in enough to place a marker. This will help you map accurately.

Scroll down to see all options.

Green areas with short-term benefits: provide limited environmental and social benefits that appear within one year of their creation. The vegetated component of these green areas includes grassy meadows and annual plants.

Short-term 📍

Green areas with medium-term benefits: offer greater environmental and social benefits compared to short-term green areas. However, these benefits only emerge 3-5 years after the area’s creation. The vegetated components of these areas can include a combination of annual plants and fast-growing trees.

Medium-term 📍

Green areas with long-term benefits: Provide broader environmental and social benefits compared to medium-term green areas, offering significant and lasting impacts. However, these benefits only

emerge within 6-10 years of the green area implementation. The vegetated component of these green area can include parks with trees and perennial plants or tree-lined avenues.

Long-term 📍

2.1 Pop-up after mapping a pin – Preferences of benefits from green areas

Listed below are eight possible benefits resulting from the creation of green areas. We ask you to select the top 3 benefits that you would like to receive in the (short/medium/long) term from this specific green area.

**Benefits from green areas**	
Air quality improvement	
Heatwave mitigation	
Biodiversity increase	
Reduction of urban noise	
Stormwater management	
Recreation opportunities	
Increase of social cohesion	
Increase of urban aesthetic with natural elements	
Food provision	

2.2 Motivation questions

Please indicate the degree of agreement with the following statements in support of the development of green areas with short-term effectiveness.

	Strongly disagree	Disagree	Neutral	Agree	Strongly agree
Short-term green areas help raise awareness about the importance of these intervention for urban sustainability.					
Short-term green areas provide a quick response to current environmental and/or social needs.					
Short-term green areas encourage the development of additional green interventions in the future.					
Short-term green areas are a reliable source of environmental and/or social benefits.					

Please indicate the degree of agreement with the following statements in support of the development of green areas with medium-term effectiveness.

	Strongly disagree	Disagree	Neutral	Agree	Strongly agree
Medium-term green areas help raise awareness about the importance of these intervention for urban sustainability.					
Medium-term green areas provide a quick response to current environmental and/or social needs.					
Medium-term green areas encourage the development of additional green interventions in the future.					
Medium-term green areas represent a fair compromise between the extent of the					

benefits offered and the time it takes for them to manifest					

Please indicate the degree of agreement with the following statements in support of the development of green areas with long-term effectiveness.

	**Strongly disagree**	**Disagree**	**Neutral**	**Agree**	**Strongly agree**
Long-term green areas help raise awareness about the importance of these intervention for urban sustainability.					
Long-term green areas reduce the need for additional investments to address environmental and/or social issues					
Long-term green areas encourage the development of additional green interventions in the future.					
Long-term green areas offer significant environmental and/or social improvements					
Long-term green areas address the needs of future generations					

---

2. **Map where you would oppose to the development of new green areas in Trento!**

Click the “short term”, “medium term”, and “long term” buttons below and place them on the map to indicate where you would you oppose to the development of new green areas that provide benefits in the short, medium, and long term. You can place as many points as you like for each type of green area.

Make sure to zoom in enough to place a marker. This will help you map accurately.

Short-term

Medium-term

Long-term

3.1 Motivation questions

Please indicate the degree of agreement with the following statements in opposition of the development of green areas with short-term effectiveness.

	Strongly disagree	Disagree	Neutral	Agree	Strongly agree
Short-term green areas require additional investments to address environmental and/or social problems.					
Short-term green areas do not provide significant environmental and/or social improvements.					
Short-term green areas do not take into account the needs of future generations.					
Short-term green areas are measures aimed at temporarily satisfying public opinion.					
Short-term green areas are not necessary.					

Please indicate the degree of agreement with the following statements in oppose of the development of green areas with medium-term effectiveness.

	Strongly disagree	Disagree	Neutral	Agree	Strongly agree

Medium-term green areas attract less public support because they do not yield immediate results.					
Medium-term green areas do not take into account the needs of future generations.					
Medium-term green areas do not provide significant environmental and/or social improvements.					
Medium-term green areas do not guarantee the achievement of the expected benefits.					
Medium-term green areas are not necessary.					

Please indicate the degree of agreement with the following statements in oppose of the development of green areas with long-term effectiveness.

	**Strongly disagree**	**Disagree**	**Neutral**	**Agree**	**Strongly agree**
Long-term green areas do not respond quickly to current environmental and/or social needs.					
Long-term green areas attract less public support because they do not yield immediate results.					
Long-term green areas do not guarantee the achievement of the expected benefits.					
Long-term green areas are not necessary.					

---

3. Sociodemographic questions

We will end the survey with some personal questions. Please remember that the answers to the survey will be treated anonymously.

1. Gender:
    - Female
    - Male
    - Other

2. Age:
    - 18-24
    - 25-34
    - 35-44
    - 45-54
    - 55-64
    - 65+

3. Education:
    - Primary school
    - Middle school
    - High school
    - Bachelor's degree
    - Master's degree
    - Doctoral degree

4. Job situation:
    - Employed
    - Unemployed
    - Student
    - Stay at home
    - Retired
    - In other conditions

Congratulations, you have completed the survey!

Thank you so much for your responses. We now have a clear understanding of your opinions and perspectives on green areas in Trento.

For any further questions regarding the survey and your data, please send an email to alessia.chelli@unitn.it.
